# A Unifying Method to Study Respiratory Sinus Arrhythmia Dynamics Implemented in a New Toolbox

**DOI:** 10.1523/ENEURO.0197-23.2023

**Published:** 2023-10-26

**Authors:** Valentin Ghibaudo, Jules Granget, Matthias Dereli, Nathalie Buonviso, Samuel Garcia

**Affiliations:** 1 Centre de Recherche en Neuroscience de Lyon, Lyon, 69500, France; 2 Centre National de la Recherche Scientifique, Lyon, 69500, France; 3 Institut National de la Santé et de la Recherche Médicale, UMRS1158 Neurophysiologie Respiratoire Expérimentale et Clinique, Sorbonne Université, Paris, 75005, France

**Keywords:** cycle-by-cycle, respiration, respiratory sinus arrhythmia, toolbox

## Abstract

Respiratory sinus arrhythmia (RSA), the natural variation in heart rate synchronized with respiration, has been extensively studied in emotional and cognitive contexts. Various time or frequency-based methods using the cardiac signal have been proposed to analyze RSA. In this study, we present a novel approach that combines respiratory phase and heart rate to enable a more detailed analysis of RSA and its dynamics throughout the respiratory cycle. To facilitate the application of this method, we have implemented it in an open-source Python toolbox called physio. This toolbox includes essential functionalities for processing electrocardiogram (ECG) and respiratory signals, while also introducing this new approach for RSA analysis. Inspired by previous research conducted by our group, this method enables a cycle-by-cycle analysis of RSA providing the possibility to correlate any respiratory feature to any RSA feature. By employing this approach, we aim to gain a more accurate understanding of the neural mechanisms associated with RSA.

## Visual Abstract

**Figure:**
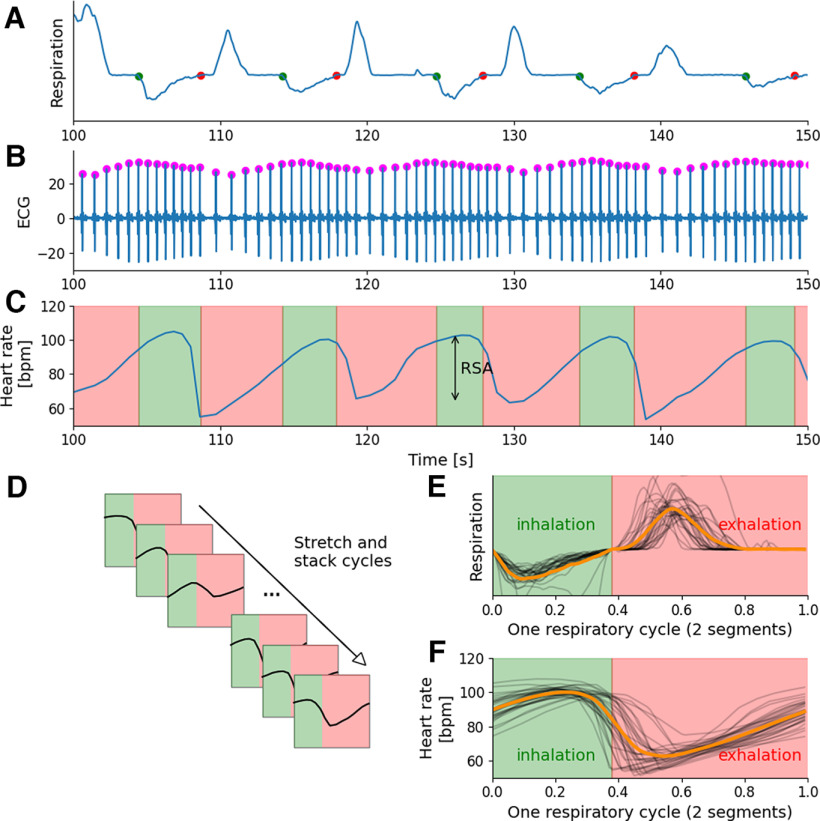

## Significance Statement

Respiratory sinus arrhythmia (RSA), the natural variation in heart rate synchronized with respiration, has been extensively studied in emotional and cognitive contexts. Various time or frequency-based methods using the cardiac signal have been proposed to analyze RSA. This work presents a novel approach that combines respiratory phase and heart rate to enable a more detailed analysis of RSA and its dynamics over time and throughout the respiratory cycle. It is implemented in an open-source toolbox that incorporates this framework in easily configurable functions and readable code.

## Introduction

Brain and body have a strong bi-directional interaction through a wide range of rhythms, explained by the plethoric literature on the topic (Thayer and Lane, 2009; Azzalini et al., 2019; Sevoz-Couche and Laborde, 2022). Thus, noninvasive recordings of neurophysiological signals are now widely used to gain new insights in cognitive neuroscience. Many toolboxes have been developed to standardize the processing of signals, such as respiration and heart signals [electrocardiogram (ECG)], to extract respiration features, heart rate variability (HRV), and the amplitude of respiratory sinus arrhythmia (RSA; Noto et al., 2018; Makowski et al., 2021; Kirk et al., 2022).

RSA, which refers to the natural variation in heart rate synchronized with respiration, has gained significant attention as a noninvasive marker of autonomic nervous system activity and cardiovascular health (Porges, 2011). Various methods have been developed characterize RSA, but most of them focused only on its amplitude (Grossman et al., 1990; Lewis et al., 2012).

Our group previously developed and used a framework to analysis neural events and its relations to the respiratory cycle (Roux et al., 2006). The main idea was to get a phase representation of the respiratory cycle as a normalized time basis, enabling standardized data collection across different subjects and facilitating the averaging of respiratory driven activity. We found particularly relevant to reuse this method to analyze RSA and its dynamic along the respiratory signal. Indeed, given the link between respiration and heart rate, it appears essential to be able to characterize both (1) the RSA dynamics (2) the respiration RSA phase-locking as a function of respiration duration and/or amplitude. For this purpose, cycle-by-cycle analysis is relevant. This has already been partly tackled by previous work (Kotani et al., 2000; Gilad et al., 2005). But, to go further, combining these approaches would be particularly useful.

For this purpose, we propose a new python toolbox, named physio, that incorporates this framework into the classical RSA extraction pipeline, aiming at enhancing the understanding of heart rate dynamics in relation to the respiratory cycle. Our toolbox provides easily configurable functions and readable code. It includes functions for processing ECG and respiratory signals providing cycle-by-cycle respiratory features. These are necessary for computing RSA. Last but not least, users have access to key parameters concerning the normal range of heart or respiratory rate, making the toolbox applicable to both humans and animals.

## Materials and Methods

### Toolbox description

#### Overview

physio was developed in Python and its well established scientific toolstack (NumPy, SciPy, pandas). Code, documentation, and tutorials are freely available here:

- Code: https://pypi.org/project/physio
- Documentation: https://physio.readthedocs.io
- Notebooks for generating figures: https://github.com/samuelgarcia/physio_benchmark

**Figure 1.**
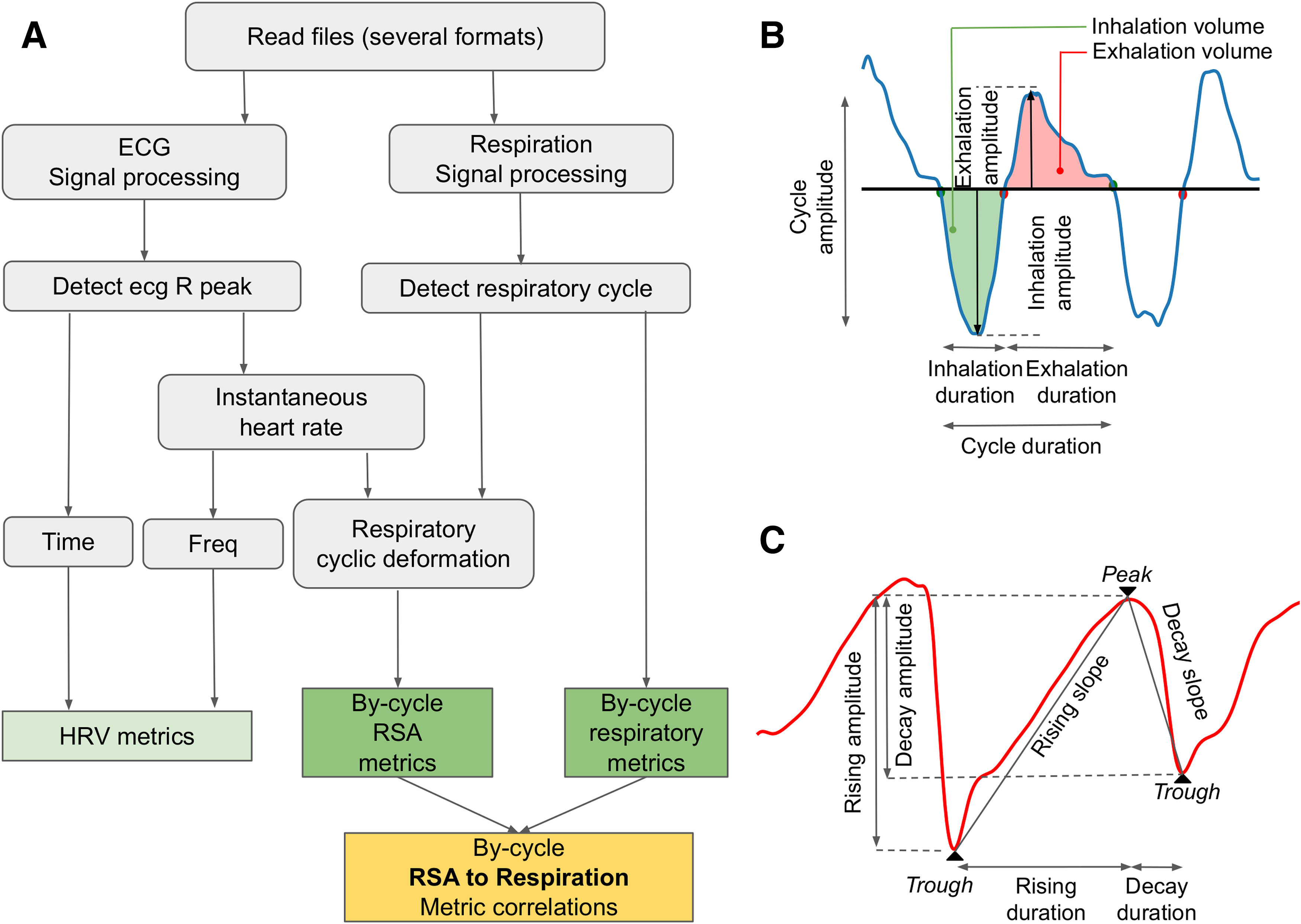
Pipeline and features extraction. ***A***, Overview of the pipeline. The dark green and yellow boxes highlight the uniqueness of the toolbox. ***B***, Respiratory features. All features are individually collected for each cycle. ***C***, RSA features detection. All features are individually collected for each cycle.

Our method requires the processing of both ECG and respiration signals to compute RSA dynamics. Our aim was to provide for the user a possibility to access to key parameters of these two processing steps through low-level functions and light readable code. Processing steps are similar for humans and animals even if key parameters could differ. In the following paragraphs, we will detail the processing steps using human model parameters (toolbox defaults). These steps are presented in a schematic view presented in Figure 1*A*. The different processing stages are as follows.

#### Respiration cycle detection (Fig. 2*A*)

**Figure 2.**
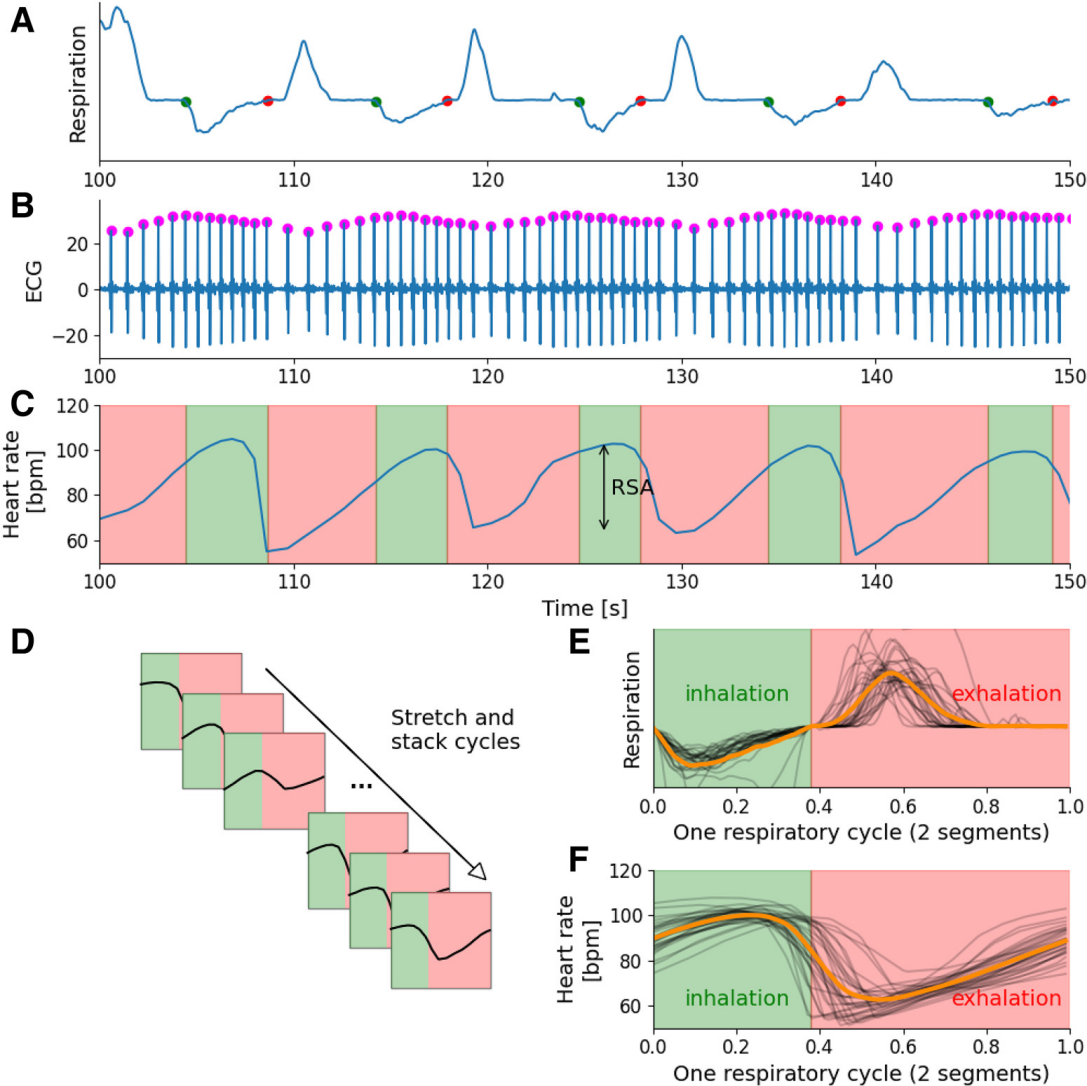
Computational procedure for RSA extraction. ***A***, Respiratory cycle detection. Baseline crossing points are detected on preprocessed signal: inhalation and exhalation starts and represented by green and red dots, respectively. ***B***, R peak detection on ECG. After ECG filtering, R peaks are detected by finding local maxima (purple dot). ***C***, Instantaneous Heart rate signal reconstruction. RR intervals in seconds are converted to beats per minute. RR intervals are interpolated on regularly sampled time series. Respiratory epochs are displayed by pink and green time zones for exhalation and inhalation respectively. ***D***, Cyclical deformation of heart rate epochs. Heart rate signals are windowed according to respiratory cycle epochs. Each epoch time axis is rescaled to a respiratory phase basis with alternating inhalation and exhalation phases. ***E***, Respiratory cycles stretched. 0: inhalation starting phase point; 0.4: inhalation-exhalation transition phase point; 1: exhalation stop phase point. The average waveform is plotted in orange. ***F***, RSA dynamics along respiratory phase. With the same process, heart rate dynamics are computed and plotted along respiratory phase. Various heart rate epochs (black traces) are averaged across cycles to get the mean RSA dynamic along the respiratory phase (orange).

The first step aims at extracting respiratory timestamps that will be used as a template to explore heart rate dynamics. Example of detection is presented in Figure 2*A* with inhalation starting times shown by green dots and exhalation starting times shown by red dots.

- Respiratory signal preprocessing: constant detrending (mean/median subtraction), filtering (low-pass, Bessel, 7 Hz) and optional temporal smoothing (Gaussian kernel, 60 ms, working also as a low-pass filter).
- Baseline estimation using signal median or signal mode.
- Respiratory cycle detection: baseline crossings were timestamped, and labeled as inhalation start if corresponding signal is decaying, or labeled as exhalation start if corresponding signal is rising.
- Computing respiration features: cycle-by-cycle respiratory features such as duration, amplitude, volume (Fig. 1*B*).
- Cleaning respiration features: remove cycle outliers due to artifacts or poor detection, using log-transform of the respiratory volume and threshold based on the median absolute deviation (MAD; 4 by default). Thus, respiratory cycles that are too short in time and/or in amplitude are removed.

#### ECG to heart rate

The second step, consisting in processing ECG to extract heart rate dynamics across time (heart rate signal), was processing as follows:

- ECG signal preprocessing: filtering (bandpass, Bessel, 5 to 45 Hz). These parameters allow for an increase in signal-to-noise ratio, signal being the R peak, noise being outer frequency components (slow drift, P and T cardiac cycle wave, line-noise).
- Normalization: median subtraction before division by median absolute deviation (MAD) which is defined by $k*median(|x_{i}-median(x)|)$ where *k* = 1.4827 and *x* corresponds to the traces values. MAD provides a dispersion statistical metric that is more robust to outliers than SDs.
- R peak detection (Fig. 2*B*, purple dots): only the traces whose amplitude is greater than an automatically calculated threshold are kept (K MAD deviations from the median). In these traces, the peaks are detected using a sliding window which finds the local maxima.
- R peak cleaning: only peaks separated by a minimum interval (400 ms by default) are kept for analysis.
- R-R intervals were computed by taking the time difference between R peak times. By default, RRI interval units (or heart periods) are converted to beats per minute (60/RRI time differences in seconds), units more commonly used in physiology. Note that at this step, RR time periods can be statistically characterized as a distribution whose position and dispersion metrics give access to time domain heart rate variability (HRV) metrics (mean, median, MAD, RMSSD, SD, coefficient of variation).
- Instantaneous heart rate signal construction by interpolation of RRI values on regularly sampled time vector (default 100 Hz). This provides an instantaneous heart rate signal (in beats per minute) or an instantaneous heart period signal (in milliseconds) depending on the preferred unit. Here, frequency components of this signal can be computed through fast Fourier transform (FFT) in ordered to compute classical frequency domain HRV metrics (low-frequency power, high-frequency power).

#### Respiratory phasing of RSA: method incorporation

In the next section we provide a brief overview of the RSA extraction pipeline implementing the new framework that is more deeply explained below, Respiratory phasing of RSA.

- Cyclical deformation of heart rate signal based on respiratory cycle timestamps.
- This heart rate signal cyclically deformed is sliced according to respiratory timestamps (Fig. 2*D*) in order to get one trace by respiratory cycle (Fig. 2*F*).
- Cycle-by-cycle feature extraction of heart rate respiratory epochs. Peak to trough amplitude differences of each heart rate epoch are computed to obtain cycle-by-cycle RSA amplitude (Fig. 1*C*). Each heart rate deformed epoch provides one amplitude value by respiratory phase bins (default is *N* = 50 bins). This phase-amplitude representation allows for computing features of RSA dynamics (Fig. 1*C*) such as rising/decaying duration, amplitude, and slope.

### Key parameters availability

The previous section outlined the primary computational steps involved in the transformation of raw signals for RSA exploration. Each major step consists of multiple substeps, and it was our intention to provide users with access to key parameters at both levels of the algorithm hierarchy. As a result, the major steps can be easily configured using a few high-level functions, using preset parameters that are appropriately adjusted for “normal” breathing humans by default, or for “normal” breathing animals such as rodents.

Depending on the experimental conditions, the respiratory features of the subjects may vary from normal to slower (e.g., slow-paced breathing) or faster (e.g., running human and/or naturally fast-breathing animals). In such cases, the traditionally set parameters of many toolboxes cannot be used, as they assume a typical respiratory frequency ranging from 0.12 to 0.40 Hz. To address this, we have made the parameters of the low-level functions accessible, allowing users to customize their analysis based on their specific experimental conditions.

### Respiratory phasing of RSA

This method is an update from several previous works (Kotani et al., 2000; Gilad et al., 2005; Roux et al., 2006). This work was initially made to deal with the rhythmic variability of respiratory cycle duration, making it impossible to average oscillatory neural patterns along respiration phase. Briefly, the time component of respiratory epochs, which can differ from trial to trial, was converted into a phase component defined as [−*π*,0] and [0,*π*] for inspiration and expiration, respectively. As opposed to time representation, the phase representation is common to all epochs regarding the phase axis. Thus, this phase representation of the respiratory cycle can be used as a normalized time basis allowing to collect results in a standardized data format across different subjects and providing a way to average oscillatory components of the activity. This is a particularly relevant method to characterize activity such as heart rate dynamics normalized on a respiratory time basis.

The original method have been upgraded with two concepts: first, the respiratory phase is scaled into [0–1] (rather than the previous [−*π*,*π*]) so that it can be divided into one or more segments. For instance, the inhalation phase can be stretched to [0–0.4] and the exhalation phase [0.4–1], this ratio can be change to fit the per subject real inhalation/exhalation duration ratio. We can also divide the respiratory cycle into more segments using extremum of inhalation or exhalation and even pauses in the respiratory phase. Example in Figure 2 are shown with two segments (inhalation/exhalation). Second, the method uses a simpler and faster linear interpolation rather than the FFT for interpolation.

This is why we reused this process as summarized in Figure 2:

- Heart rate signal is segmented into successive respiratory cycles with alternating inspiration and expiration windows.
- Each window is re-sampled with a linear interpolation that keeps the same number of bins per segment. This width is a configurable parameter that depends on the desired precision (default is *N* = 40 bins).
- The phases of the inspiration and expiration windows are taken linearly between [0,0.4] and [0.4,1]. This latter parameter is configurable by the user.

Thus, instantaneous heart rate time series is re-scaled to a respiratory phase basis with alternating inhalation and exhalation phases, allowing an acute dynamic description of heart rate according to respiratory phase. See documentation of the toolbox for examples.

### Datasets

We tested our tools on two datasets coming from two free-breathing models: human and rodents (rat). Datasets are available on the Zenodo platform at https://zenodo.org/record/8019849.

#### Human dataset

Human dataset consisting of 15 healthy adults subjects (age: 30.9 ± 9.5 years old). All participants gave informed consent to take part to the study, and all experiments were approved by the national French committee (CPP number 4090). They were sitting quietly and instructed just to relax. Recording lasted 5 min.

- Respiration signal was recorded from a nasal sensor (Sensortechnics GmbH) at a sampling rate of 1000 Hz, amplified by actiCHamp Plus amplifier (Brain Products GmbH).
- ECG signal was recorded from three skin electrodes (right forearm, left forearm, left iliac region), at a sampling rate of 1000 Hz (same amplifier).

#### Animal dataset

Animal dataset consisting of eight healthy adults rodents (rats). The experiments were carried according to the ethical guidelines of the European Communities Council Directive of November 24, 1986 (86/609/EEC), as well as the approval 16 979 of the Lyon 1 University CEEA-55 ethical committee and of the Ministry of Higher Education, Research and Innovation. Recording lasted 5 min during which they were freely behaving. Respiration signal and ECG were recorded from a thoraco-abdominal telemetric jacket (DECRO, by Etisense). Recordings were performed at a sampling rate of 500 Hz, amplified by Etisense acquisition unit (Etisense).

## Results

**Figure 3.**
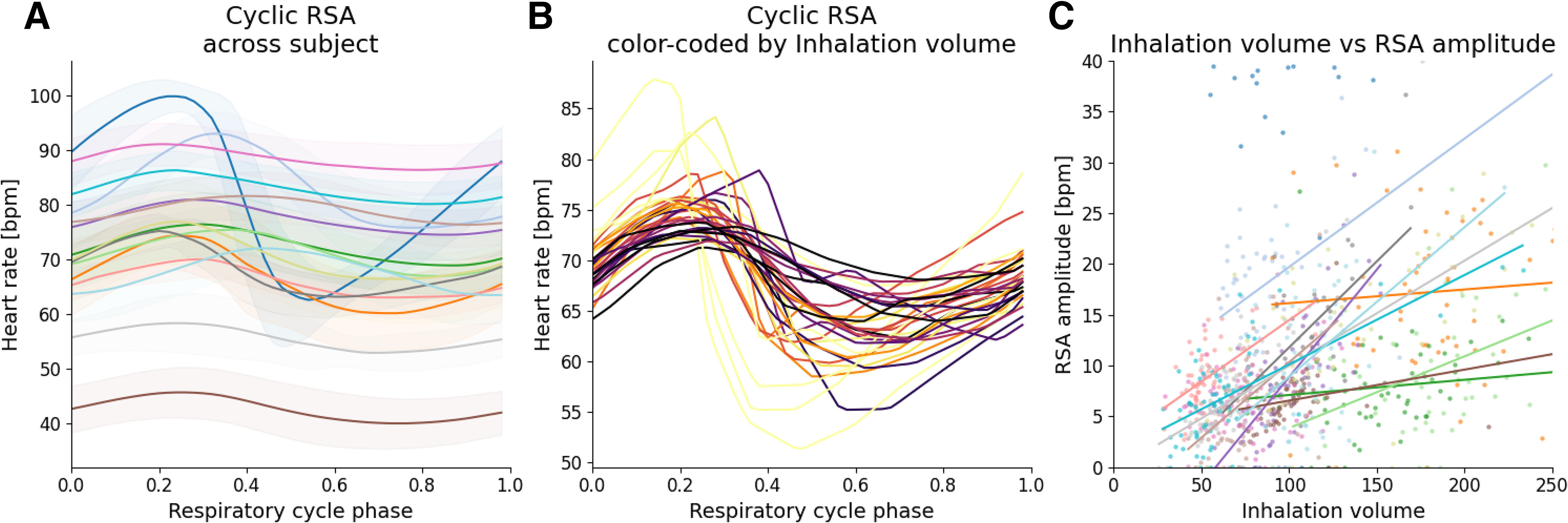
Some examples of respiratory phased RSA use. ***A***, Intersubject variability of the mean RSA. Subjects are color coded. Shades correspond to 1 SD. ***B***, Intrasubject variability of the RSA. Cycle-by-cycle RSA of one subject are represented. Color palette, from dark to light warm colors, encodes the amplitude of the corresponding inhalation (from dark to yellow). The subject corresponds to the one colored in dark gray in Figure 1*A*. ***C***, RSA to inhalation volume correlations. Only significant slopes are represented.

**Figure 4.**
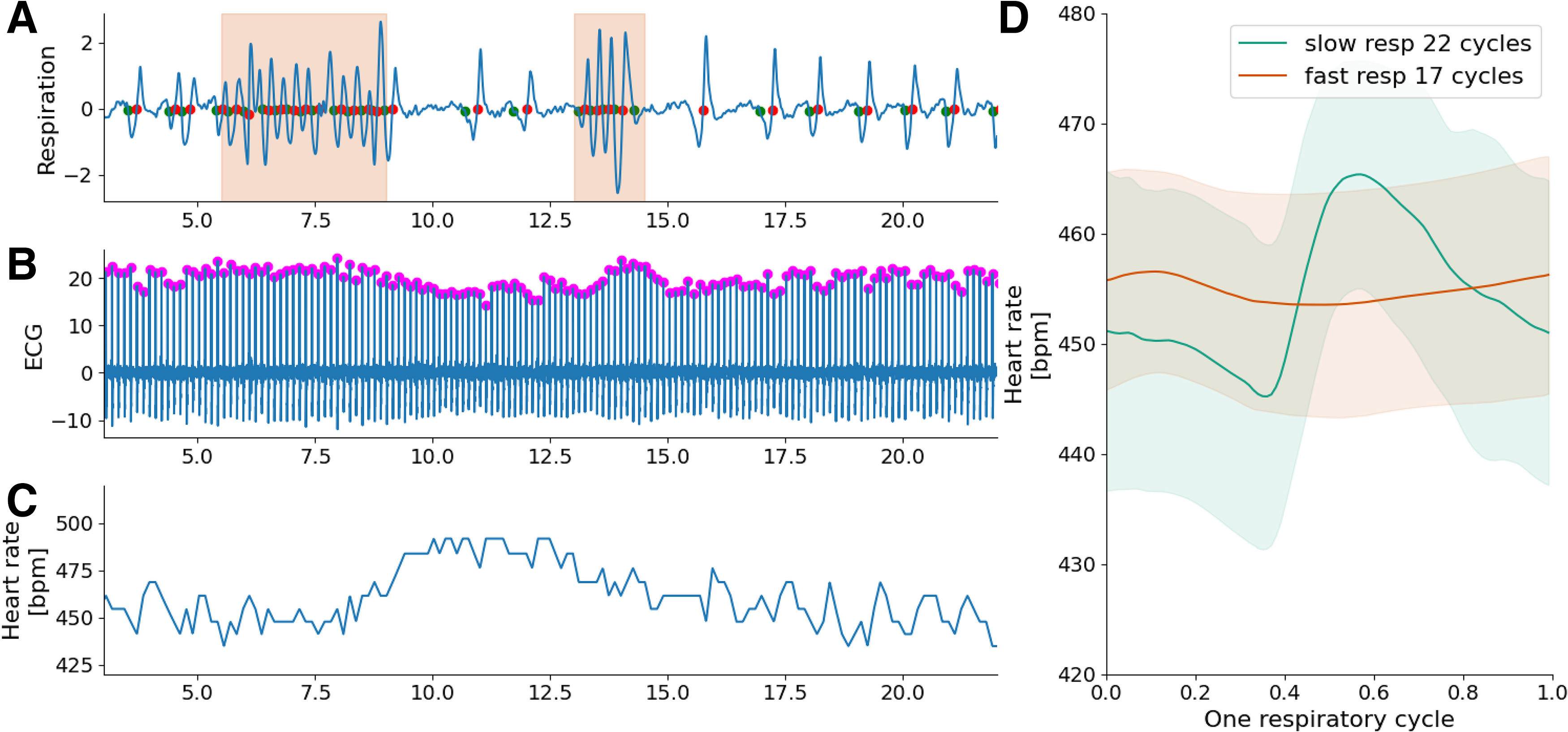
Examples of processing rat dataset. ***A***, Respiratory signal. Fast breathing periods are displayed by a shadow color zone. ***B***, ECG signal. ECG peaks have been detected thanks to rodent preset of parameters. ***C***, Heart rate. ***D***, Respiratory phased RSA average. Average of all RSA cycles while splitting the respiratory cycle in “fast” (<600 ms) and “slow” (>600 ms). Shadow represents 1 SD on each side.

We tested our tools on both human and animal datasets. Figures 3 and 4 presents some examples of computations that can be obtained thanks to the toolbox. More examples are presented in attached Jupyter notebooks.

### Humans

The following outputs were obtained using the default parameters.

Cyclically deformed and sliced heart rate signal process provides a 2D array containing stacked magnitude heart rate according to respiratory phase * respiratory cycle data from which are computed RSA features. This can be used to get cycle-by-cycle dynamics and also the averaged heart rate dynamic across several respiratory cycle (Fig. 2*F*, orange trace) and the process can be iterated over subjects to display average dynamics by subject (Fig. 3*A*). On this latter figure, note the variability in amplitude and phase of the modulation of heart rate according to the subjects (colors coded). Figure 3*B* allows to visualize the intrasubject variability of RSA dynamic in term of its phasing and its amplitude.

Then, two major cycle tables (also known as dataframes) can be obtained: one for cycle-to-cycle respiration features and the other for cycle-to-cycle RSA features. Both sharing the same number of rows (respiratory cycles), they can be concatenated to correlate cycle by cycle respiration to RSA features. Then, it is possible to correlate RSA features to respiration features to explore effects of respiration cycle duration-derived features or respiration cycle amplitude-derived features. Correlations could be then computed to explore, for instance, intersubject variability as shown in Figure 3*C* where we can see different regression slopes between subjects when exploring the RSA amplitude according to the inhalation volume.

### Animals

Switching from human to animal default preset parameters provides the same outputs. Figure 4 presents an example of processing of rat data. We can see in Figure 4*A* some examples of detection of different types of respiratory cycles (slow or fast cycles). Figure 4*C* displays the instantaneous heart rate computed thanks to the previous detection of ECG peaks shown in Figure 4*B*. Note the RSA frequency component in the heart rate that is well correlated to the respiratory signal. Cycle-to-cycle analysis allows separating RSA dynamics according to breathing cycle duration: slow respiratory cycles being correlated to larger amplitude modulation of RSA compared with fast respiratory cycles, as shown in Figure 4*D*.

## Discussion

The physio package provides a new open-source python toolbox aggregating tools for processing ECG and respiratory signals to accurately characterize the respiratory sinus arrhythmia phenomenon.

RSA, the natural variation in heart rate synchronized with respiration, is one form of cardio-respiratory coupling, physiologically independent of another phenomenon, the cardio-respiratory synchronization (Bartsch et al., 2012). RSA has been the subject of numerous studies investigating the role of vagal tone under various emotional or cognitive experimental conditions. However, the computation of RSA amplitude, which serves as a potential indicator of vagal tone (Song and Lehrer, 2003; Porges, 2011), has been a matter of debate throughout history (Grossman et al., 1990; Lewis et al., 2012). Despite methodological variations, the literature has concluded that the different methods are equivalent (Grossman et al., 1990), leaving it to the users to choose the method that best suits their research needs. Additionally, certain studies have highlighted the varying phase patterns of the RSA phenomenon during different breathing regimes (Kotani et al., 2000; Gilad et al., 2005). This is corroborated with a more recent work that evidenced the difference of vagal tone during inhalation compared with exhalation phase (Marmerstein et al., 2021). Therefore, the physio toolbox aims at providing a set of easily readable and configurable functions to explore both RSA amplitude and dynamics, applicable to various breathing regimes and models (e.g., normal breathing humans, slow-paced breathing humans, fast-paced breathing humans, rodents, etc.).

The main computational steps involve: (1) computing the ECG to extract the instantaneous heart rate time series (HRV metrics can also be obtained); (2) processing the respiratory signal to detect respiratory cycles and extract respiratory features; and (3) cyclical deformation of heart rate time series on the basis of respiratory cycle time to extract cycle-by-cycle RSA amplitude modulation features in parallel with RSA dynamic features, a process adapted from a previously published method (Roux et al., 2006). These major steps are implemented through high-level functions that can be configured by the user using preset parameters. The default configuration is set for processing normal human data, but it can be adjusted for animal data as well. Customization of key computational parameters is possible through the use of easily configurable low-level functions, providing flexibility to accommodate specific experimental tasks or models that involve nonstandard physiological variables.

While there are other well-established software tools like Neurokit2, BioSPPy, that propose methods for analyzing heart and respiratory signals together, physio introduces some novel elements. It offers a cycle-by-cycle RSA exploration with innovative metrics, in addition to the possibility of correlating any dynamic phenomena to respiratory phase. Going back to the Figure 1*A*, the dark green and yellow boxes highlight the uniqueness of the toolbox.

Thus, we provided human and rodent datasets from which figures and documentation have been supplied. In addition to the precise visualization of RSA dynamics along respiration phase across different subjects (Fig. 3*A*), we illustrated the possibility to correlate RSA features to respiratory features, enabled by the cycle-by-cycle analysis provided in the final cycle tables (Fig. 3*B*). As the examples displayed in Figure 3*C* illustrate, we can see the different regression slope when plotting RSA amplitude according to inhalation volume. One could speculate that this result is because of differences in autonomic tone between the subjects as this could modulate the relationship between respiratory cycle and RSA amplitude. Furthermore, examples of RSA dynamics along respiration phase shown in Figure 3*A* emphasize the intersubject variability concerning the amplitude and the phase of dynamical changes of heart rate. This visualization also highlights the pronounced steepness of the decaying phase of heart rate compared with its rising phase, around the inhalation to exhalation phase transition (see also Fig. 2*C*). Deep exploration of RSA dynamics is essential for uncovering the underlying neural mechanisms responsible for this phenomenon.

Thus, we firmly believe that to accomplish the objective of capturing the maximum amount of information that RSA could bring to cognitive neurosciences, it is essential to accurately extract both respiratory and ECG features for analyzing RSA amplitude in relation to the respiratory phase. Only a detailed cycle-by-cycle analysis of RSA dynamics offered by the physio toolbox can provide the relevant framework necessary for understanding the whole phenomenon of cardio-respiratory coupling.

In conclusion, physio offers valuable tools for comprehending heart rate dynamics in relation to the respiratory cycle in diverse models. It aims to achieve this by providing easily configurable functions encoded in a concise and readable algorithm, enabling researchers to adapt their analysis strategies to their specific data conditions.

## Synthesis

Reviewing Editor: Silvia Pagliardini, University of Alberta Decisions are customarily a result of the Reviewing Editor and the peer reviewers coming together and discussing their recommendations until a consensus is reached. When revisions are invited, a fact-based synthesis statement explaining their decision and outlining what is needed to prepare a revision will be listed below. The following reviewer(s) agreed to reveal their identity: Ronny Bartsch.

In “A unifying method to study Respiratory Sinus Arrhythmia dynamics implemented in a new toolbox” the authors describe software that they have developed to quantify the correlative signals between heart-rate and breathing, or respiratory sinus arrhythmia (RSA).

The manuscript is a methodological description of the tools that are incorporated into a Python-based suite of tools called “physio” and available at https://pypi.or/project/physio. The tools included in physio have the potential to be very useful in the context of cardio-respiratory data analysis. The literature cited is appropriate for the topic of RSA and is current.

The manuscript is a good example of an Open Source Tools and Methods paper. The authors use two example datasets (one human, one rodent) to validate application of the tools. The documentation available on the website provides a comprehensive overview of physio with tutorial examples, which are very useful for learning how to use physio.

In my opinion, this is a very useful addition to the software armamentarium of cardio-respiratory physiologists and can serve as a foundation for a more extensive set of software tools dedicated to cross-correlation of cardiac and respiratory signals.

While I appreciate the effort to provide open-source code for physiological analysis and help scientists easily obtain RSA estimates for their research, the manuscript requires a significant revision before publication. As a general comment, I am missing a paragraph discussing already available open-source HRV and RSA analysis packages. For example, the authors cite Makowski et al. (the developer of NeuroKit2) but do not relate to their package, which is doing similar things as physio. This would help researchers/users to learn more about the advantages of “physio” compared to other open-source software. For the revision, authors should also consider thorough editing as the current version has quite a few typos and mistakes in English grammar.

I have some minor concerns/queries that I include below:

1. I like the use of “plethoric” in the first sentence of the

Introduction but “litterature” should be “literature” and the sentence seems incomplete. Perhaps the authors meant, “explained by the plethoric literature on the topic.” as this would make a complete sentence?

2. Throughout. I believe Python, NumPy, SciPy are capitalized as

proper nouns and suggest that this should be the standard in the

manuscript.

3. Methods. “Next paragraphs will detail processing steps configured using human model parameters (default of the toolbox)” is awkward and I would suggest rewriting to read, “In the following paragraphs, we will detail the processing steps using human.... (toolbox defaults).”

4. “centering” as used in the methods is what is typically referred to as “detrending” in the signal theory field.

5. The authors should explain why they used a 60 millisecond Gaussian kernel.

6. “due artifact” should be changed to “due to artifacts...”

7. Page 3, top, Normalization bullet point. “substraction” should be “subtraction” and there is an extra closing parenthesis at the end of the line.

8. Bullet point for R peak cleaning. The description would be easier to read as, “Only peaks separated by a minimum interval (400 ms by default) are kept for analysis.

9. Next bullet point should read, “R-R intervals were computed...”

10. “beat per minute” should be changed to “beats per minute”.

11. The section on Respiratory phasing of RSA. The description could be re-written to say, “In the next section we provide a brief overview of the RSA extraction pipeline...”

12. The section on Cycle-by-cycle feature extraction of hear rate respiratory epochs. The first sentence could be changed to, “Peak to trough amplitude differences of each heart rate epoch are computed to obtain cycle-by-cycle RSA amplitude (Figure 1C).” Also, “provide one amplitude value...” should be “provides one...”

13. Respiratory phasing of RSA section. “several previous work” should be changed to “several previous works”.

14. In the second bullet point for this section, please change “keep” to “keeps” and “bin” to “bins”.

15. Section 2.4, Dataset (should be “Datasets”). Change “Human dataset consist in 15...” to “Human dataset consisting of...” “french” should be capitalized to “French”. In the animal dataset section, make a similar change to, “Animal dataset consisting of...”

16. Sect 3, Results. Please change “The figures 3 and 4...” to “Figures 3 and 4 presents...” Attached notebooks (I assume Jupyter notebooks” are mentioned but were not part of the submission. A simple link to the GitHub repository including those notebooks would be a useful addition for the reader.

17. In Humans. “(colors encoded)” should read “(color encoded)”. I am not sure what is intended by “confront cycle by cycle”. Do the authors mean “correlate cycle-by-cycle”? Last sentence, “slope” should be “slopes”.

18. In Animals. Add “an” to make “presents an example of processing...”

19. Discussion. Paragraph 2, “3) cyclical deformation of heart rate time series by respiratory cycle time basis to extract cycle by cycle...” might be clearer if written as “3) cyclical deformation of heart rate time series on the basis of respiratory cycle time to extract cycle-by-cycle...”

20. “Thus, we provided human and rodent datasets...” (plural rather than singular). Also, in this p ragraph, “One could speculate this result coming from the various vagal tone of the subjects differently mediating the relation between respiratory regime and RSA amplitude.”

would be clearer as, “One could speculate that this result is due to differences in autonomic tone between the subjects as this could modulate the relationship between respiratory cycle and RSA

amplitude.” Or something similar but in keeping with the authors’

intended idea.

21. Figure 1 (legend). “individually collected” instead of “individual collected”.

Other comments as they appear in the manuscript:

Page 2 (respiratory cycle detection):

- please explain why baseline crossings are used instead of applying a Hilbert transform and the analytic signal

approach to estimate the respiratory phase

- please define MAD

Page 3:

- R-peak detection seems to be very simple, but is it robust towards artifacts such as baseline shifts? For example, the Pan-Tompkins

has several pre-processing steps to deal with this. Also, when studying patients or the elderly, there could be a significant amount of ectopic

beats. Can physio detect and remove them automatically?

Fig. 1A - Read files (several formats)

Fig. 3

(A) what are the shades around the curves (standard deviation? standard error?)

(B) which subject of (A) is shown here?

(C) typo: Inhalation volume; I find this figure to be a bit messy. Can the authors show just a few representative examples?

Discussion

- to my knowledge, one of the first works to report a difference in vagal tone during inhalation compared to exhalation was by Song and Lehrer (Appl Psychophysiol Biofeedback 28:13-23, 2003).

- RSA is only one form of cardio-respiratory coupling (another prominent form is cardio-respiratory synchronization, and often researchers confuse between both);

I think it would be interesting for the reader if authors mention that RSA and cardio-respiratory synchronization are two different independent physiological phenomena (see, e.g., Bartsch et al. PNAS (2012, vol. 109(26), pp. 10181-10186)

- the authors should discuss whether there are limitations of their algorithm when analyzing long-term physiological data (for example, from sleep-lab recordings or 24-h ECGs)

## Author Response

Reply to reviews

July 27, 2023

1 Major point

Review : As a general comment, I am missing a paragraph discussing already available open-source HRV and RSA analysis packages. For example, the authors cite Makowski et al. (the developer of NeuroKit2) but do not relate to their package, which is doing similar things as physio. This would help researchers/users to learn more about the advantages of “physio” compared to other open-source software.

Answer : We do agree. We added an explanation at the end of the second paragraph of the discussion providing the main innovations of the toolbox compared to others, added to a mention in the legend of the figure 1A that highlight its uniqueness with the dark green and yellow boxes. Besides, the main objective of this work is to provide a new method for exploring RSA, helped by a toolbox. So, we didn’t feel the need to extensively benchmark against other software tools on all other aspects, such as the accuracy of respiratory cycle detection and ECG peak detection.

2 Minor points

1. Review : I like the use of “plethoric” in the first sentence of the Introduction but “litterature” should be

“literature” and the sentence seems incomplete. Perhaps the authors meant, “explained by the plethoric literature on the topic.” as this would make a complete sentence?

Answer : Corrected

2. Review : Throughout. I believe Python, NumPy, SciPy are capitalized as proper nouns and suggest that this should be the standard in the manuscript.

Answer : Corrected

3. Review : Methods. “Next paragraphs will detail processing steps configured using human model pa- rameters (default of the toolbox)” is awkward and I would suggest rewriting to read, “In the following paragraphs, we will detail the processing steps using human.... (toolbox defaults).”

Answer : Corrected

4. Review : “centering” as used in the methods is what is typically referred to as “detrending” in the signal theory field.

Answer : We do agree. Corrected by “constant detrending” (constant being the mean value or the median)

5. Review : The authors should explain why they used a 60 millisecond Gaussian kernel.

Answer : Indeed, gaussian filter works as a low-pass with its smoothness depending on the duration of the time window parameter. Moreover, it is sometimes convenient for the user to find the right parameters in the time-domain (gaussian) rather than in the frequency domain (bessel). Empirically, 60 ms was the best gaussian time window to remove short fluctuations of the signal that could increase probability of noisy detected respiratory cycles, while not much jittering phase. Note that, as mentioned in the manuscrit, this time-domain smoothing is optional. We shortly precised it in the manuscrit : “ optional temporal smoothing (Gaussian kernel, 60ms, working also as a low-pass filter)”

6. Review : “due artifact” should be changed to “due to artifacts...” Answer : Corrected

7. Review : Page 3, top, Normalization bullet point. “substraction” should be “subtraction” and there is an extra closing parenthesis at the end of the line.

Answer : Corrected (sadly, “soustraire” in french contains the ‘s’)

8. Review : Bullet point for R peak cleaning. The description would be easier to read as, “Only peaks separated by a minimum interval (400 ms by default) are kept for analysis.

Answer : Corrected

9. Review : Next bullet point should read, “R-R intervals were computed...” Answer : Corrected

10. Review : “beat per minute” should be changed to “beats per minute”.

Answer : Corrected

11. Review : The section on Respiratory phasing of RSA. The description could be re-written to say, “In the next section we provide a brief overview of the RSA extraction pipeline...”

Answer : Corrected

12. Review : The section on Cycle-by-cycle feature extraction of hear rate respiratory epochs. The first sentence could be changed to, “Peak to trough amplitude differences of each heart rate epoch are computed to obtain cycle-by-cycle RSA amplitude (Figure 1C).” Also, “provide one amplitude value...” should be

“provides one...” Answer : Corrected

13. Review : Respiratory phasing of RSA section. “several previous work” should be changed to “several previous works”.

Answer : Corrected

14. Review : In the second bullet point for this section, please change “keep” to “keeps” and “bin” to “bins”.

Answer : Corrected

15. Review : Section 2.4, Dataset (should be “Datasets”). Change “Human dataset consist in 15...” to “Human dataset consisting of...” “french” should be capitalized to “French”. In the animal dataset section, make a similar change to, “Animal dataset consisting of...”

Answer : Corrected

16. Review : Sect 3, Results. Please change “The figures 3 and 4...” to “Figures 3 and 4 presents...” Attached notebooks (I assume Jupyter notebooks” are mentioned but were not part of the submission. A simple link to the GitHub repository including those notebooks would be a useful addition for the reader. Answer : Corrected. Link to Github repository including jupyter notebooks is already provided in the 2.1

Section

17. Review : In Humans. “(colors encoded)” should read “(color encoded)”. I am not sure what is intended by “confront cycle by cycle”. Do the authors mean “correlate cycle-by-cycle”? Last sentence, “slope” should be “slopes”.

Answer : Corrected

18. Review : In Animals. Add “an” to make “presents an example of processing...” Answer : Corrected

19. Review : Discussion. Paragraph 2, “3) cyclical deformation of heart rate time series by respiratory cycle time basis to extract cycle by cycle...” might be clearer if written as “3) cyclical deformation of heart rate time series on the basis of respiratory cycle time to extract cycle-by-cycle...”

Answer : Corrected

20. Review : “Thus, we provided human and rodent datasets...” (plural rather than singular). Also, in this p ragraph, “One could speculate this result coming from the various vagal tone of the subjects differently mediating the relation between respiratory regime and RSA amplitude.” would be clearer as, “One could speculate that this result is due to differences in autonomic tone between the subjects as this could modulate the relationship between respiratory cycle and RSA amplitude.” Or something similar but in keeping with the authors’ intended idea.

Answer : Corrected

21. Review : Figure 1 (legend). “individually collected” instead of “individual collected”.

Answer : Corrected

Other comments as they appear in the manuscript:

1. Page 2 (respiratory cycle detection):

Review : please explain why baseline crossings are used instead of applying a Hilbert transform and the analytic signal approach to estimate the respiratory phase

Answer : Respiratory signal not being a true sinusoidal signal (the two phases of the respiratory cycle are unbalanced, inhalation being shorter than exhalation), phase angles of the analytic signal of the Hilbert transform leads to an estimation of the respiratory phase that is locally non-linear. In our context, the phase is seen as a local clock (at the cycle scale), that ensures the inhalation and exhalation being com- puted separately.

Review : please define MAD

Answer : An equation and some explanations were added.

2. Page 3:

Review : R-peak detection seems to be very simple, but is it robust towards artifacts such as baseline shifts? For example, the Pan-Tompkins has several pre-processing steps to deal with this. Also, when studying patients or the elderly, there could be a significant amount of ectopic beats. Can physio detect and remove them automatically?

Answer : It already exist toolboxes that implement and compare several R peak detectors like Neurokit2 (https://github.com/neuropsychology/NeuroKit) and py-ecg-detector (https://github.com/berndporr/py- ecg-detectors). However, our method is simple, robust, and tested against public datasets. However, integrating several R peak detector algorithms is a future perspective of the toolbox.

Review : Fig. 1A - Read files (several formats) Answer : Corrected

Fig. 3

Review : (A) what are the shades around the curves (standard deviation? standard error?) Answer : We added this information : “Shades correspond to one standard deviation.” Review : (B) which subject of (A) is shown here?

Answer : We added this information : “The subject corresponds to the one colored in dark grey in figure 1a.”. Contrary to the final figure 3, the public notebook (https://github.com/samuelgarcia/physio_benchmark/ blob/main/figure_3_examples.ipynb) contains the legend with subject numbers. Results displayed in figure

3B correspond to subject S10 (dark grey in figure 3A).

Review : (C) typo: Inhalation volume; I find this figure to be a bit messy. Can the authors show just a few representative examples?

Answer : Typo mistake corrected. However, figure 3C aims more to represent the large inter-subject variability of RSA, inhalation volume, and moreover RSA to inhalation volume correlation, than presenting a true and clear result. This is why we think useful to keep such a representation.

Discussion

Review : to my knowledge, one of the first works to report a difference in vagal tone during inhalation compared to exhalation was by Song and Lehrer (Appl Psychophysiol Biofeedback 28:13-23, 2003).

Answer : Indeed, we therefore added a citation of this work

Review : RSA is only one form of cardio-respiratory coupling (another prominent form is cardio-respiratory synchronization, and often researchers confuse between both); I think it would be interesting for the reader if authors mention that RSA and cardio-respiratory synchronization are two different independent physiological phenomena (see, e.g., Bartsch et al. PNAS (2012, vol. 109(26), pp. 10181-10186)

Answer : Indeed. Thank you for this comment. We mentioned it at the end of the first phrase of the first paragraph of the discussion : “RSA, the natural variation in heart rate synchronized with respiration, is one form of cardio-respiratory coupling, physiologically independent of the well described cardio-res- piratory syn- chronization phenomenon (Bartsch et al. [2012])”

Review : the authors should discuss whether there are limitations of their algorithm when analyzing long-term physiological data (for example, from sleep-lab recordings or 24-h ECGs)

Answer : We didn’t consider such limitation because, as long as the data are of sufficient quality, algorithm should work. To our opinion, it is up to the researcher using the tool to interpret its results regarding such a temporality, like, for example, the circadian effects on RSA. Moreover, our toolbox, by design, with the cycle by cycle approach, provides all the materials to study RSA on such long temporality. Besides, we are currently using it (for an unpublished work) to analyze clinical datasets lasting for several days.

Note to the reviewers : We also added a mention in the “Fundings” section (which was “Acknowledgements” before) to Neurodis foundation that allowed us to get some materials for data recording.
